# Study on Manufacturing Technology of Ultra-Thin/Narrow Bonding Cu Strip for Electronic Packaging

**DOI:** 10.3390/mi14040838

**Published:** 2023-04-12

**Authors:** Jun Cao, Junchao Zhang, Baoan Wu, Huiyi Tang, Yong Ding, Kexing Song, Guannan Yang, Chengqiang Cui

**Affiliations:** 1 School of Mechanical and Power Engineering, Henan Polytechnic University, Jiaozuo 454000, China; zjc_xlyx@sina.com; 2 Chongqing Materials Research Institute Co., Ltd., Chongqing 400700, China; wubaoan@163.com (B.W.);; 3 Zhejiang Tony Electronic Co., Ltd., Huzhou 313000, China; 4 HeNan Academy of Sciences, School of Mechatronics Engineering, Luoyang 471000, China; 5 State Key Laboratory of Precision Electronic Manufacturing Technology and Equipment, Guangdong University of Technology, Guangzhou 510006, China

**Keywords:** rolling, annealing, bonding Cu strip, tensile strength, elongation, resistivity

## Abstract

The performance of rolling parameters and annealing processes on the microstructure and properties of Cu strip were studied by High Precision Rolling Mill, FIB, SEM, Strength Tester, and Resistivity Tester. The results show that with the increase of the reduction rate, coarse grains in the bonding Cu strip are gradually broken and refined, and the grains are flattened when the reduction rate is 80%. The tensile strength increased from 248.0 MPa to 425.5 MPa, while the elongation decreased from 8.50% to 0.91%. The growth of lattice defects and grain boundary density results in an approximately linear increase in resistivity. With the increase of annealing temperature to 400 °C, the Cu strip recovers, and the strength decreased from 456.66 MPa to 220.36 MPa while the elongation rose from 1.09% to 24.73%. The tensile strength and elongation decreased to 192.2 MPa and 20.68%, respectively, when the annealing temperature was 550 °C. The trend of yield strength of the Cu strip was basically the same as that of tensile strength. The resistivity of the Cu strip decreased rapidly during a 200~300 °C annealing temperature, then the trend slowed, and the minimum resistivity was 3.60 × 10^−8^ Ω·m. The optimum tension range annealing was 6–8 g; less or more than that will affect the quality of the Cu strip.

## 1. Introduction

The function of bonding materials is to link the silicon chip electrodes with the lead frame exterior lead terminals so as to achieve the purpose of information exchange, power transmission, and heat dissipation between the chip and the integrated circuit, which is the core of the whole microelectronic packaging process [1,2]. The quality and performance of bonding materials directly affect the quality of semiconductors and the reliability of integrated circuits [3,4]. Au bonding wire is the earliest used, with high mechanical strength, oxidation resistance, a simple bonding process, and other advantages. However, Au and Al are prone to produce harmful intermetallics, and the price of Au has risen sharply in recent years, resulting in an annual decline in the market share of Au bonding wires [5,6]. Although adding other elements to Au can improve the binding performance of Au alloy wires, the limited cost reduction limits its wide application in the microelectronic packaging market [7,8]. Ag bonding wire attracts the attention of scholars because of its excellent electrical and thermal conductivity and lower price than Au. Ag has the potential to adapt to the development trend of high integration and high-speed microelectronic packaging and is considered to be a promising bonding material [9,10]. They have not been widely used as there are still some reliability problems with Ag bonding lines [11].

As an alternative line, the Cu bonding wire has higher conductivity, thermal conductivity, and lower cost than Au and has good electrical connection potential in high power and high integration electronic devices [12,13,14,15,16,17]. Compared with Au wire, Cu wire has better electrothermal performance, higher pull force, and loop stability, allowing for thinner wires to fit a smaller pad size, and the growth rate of harmful intermetallics in Cu-Al is less than one-fifth of that in Au-Al, which greatly improves chip lifetime and reliability [18,19,20]. As Cu has higher shear and tensile strength than Au, it has better bonding efficiency during the packaging of microspacing devices [21,22,23]. The production cost of Cu bonding can be significantly reduced by optimizing and improving the manufacturing process [24]. During the bonding process, intermetallics seldom occur at the Cu-Al interface due to the low mutual diffusion rate between Cu and Al, and the bond point of Cu may have a higher durability than the Au [25,26]. Insulator coating on bare Cu conductors prevents short circuit problems of conductors and shows good joining and reliability during use, providing a potential solution for fine and ultra-fine-spacing conductor joining [27], but there are still shortcomings in high-power packaging.

**Figure 1:**
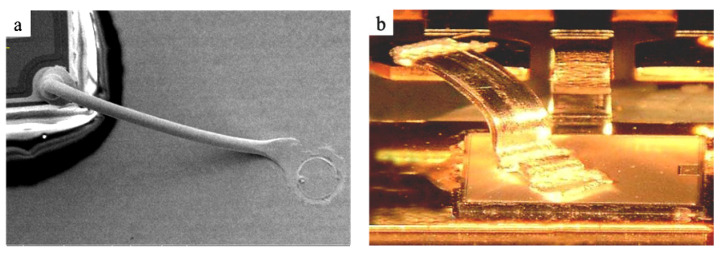
Bond alloy wire/strip contrast: (**a**) bond wire and (**b**) bond strip.

Traditional wire bonding materials are usually made of micro wire with circular cross-sections by drawing, which bonding effect is shown in Figure 1a; they are widely used in the market due to their wide variety of materials and mature manufacturing technology. However, it also has disadvantages, such as low load-carrying current and poor heat dissipation, which cannot meet the requirements of high-power packaging. So many scholars have begun to turn their attention to the research of metal bonding strips with high strength and reliability. Miniature metal narrow strips with a thickness of less than 0.1 mm and a width of less than 2 mm for electronic packaging are referred to as bonding metal strips in the standard [28]. Compared with traditional bonding wires, bonding Cu strips have the advantages of high strength, high current carrying capacity, and good heat dissipation, which can meet the requirements of microwave and high-power devices. Moreover, due to the differences in bonding methods, bonding Cu strips have a larger connection area to the substrate when bonding, providing a more reliable connection effect, as shown in Figure 1b. Therefore, research on bonding alloy strips is of great significance to the electronic industry.

However, the above research mainly focused on the composition of bonding materials, and the research on new bonding Cu strips is less involved. This paper studied the deformation behavior and annealing effect on the properties of bonding Cu strips during processing, explored the processing technology, and provided a theoretical basis for the manufacturing of bonding Cu strips.

## 2. Test Materials and Methods

### 2.1. Test Materials and Equipment

The test material is a microfine Cu wire of φ57 µm and φ35 µm; both of them were processed by a multi-stage drawing of φ8mm pure Cu rod (purity higher than 99.9997%). Specification 1 is a hardened Cu wire that has not been annealed after wire drawing, while Specifications 2 and 3 have been annealed under different annealing parameters and have slightly different strengths and elongations. Their properties are shown in Table 1 and Table 2. The equipment used in the test is shown in Table 3.

### 2.2. Test Method

**Figure 2:**
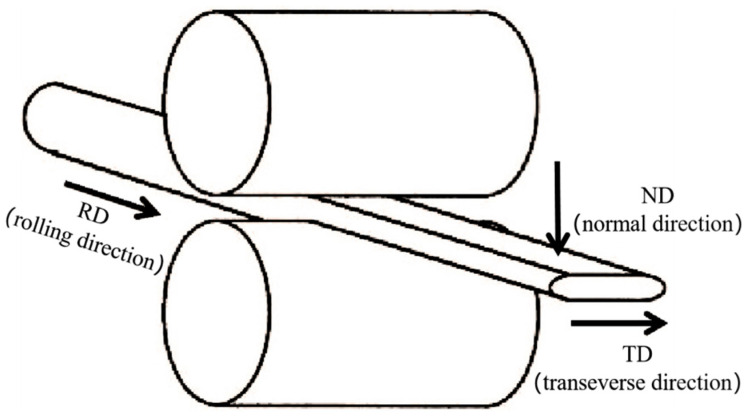
Cu wire rolling diagrammatic sketch.

The change of size of Cu wire during the rolling process varies in all three directions, as shown in Figure 2. The φ57 µm Cu wires of specifications 1, 2, and 3 were rolled into Cu strips with the same reduction ratio (60%) and studied the performance changes of different Cu wires. The φ57 µm Cu wire of Specification 2 was rolled through different reduction rates (40%, 50%, 60%, 70%, 80%). Then its tensile strength and elongation were measured with a sample 100 mm long, and the speed of the tensioner was set to 20 mm/min. Take a 1000 mm long Cu strip and measure the resistance value *R* on the resistance tester, and then calculate the Cu strip resistivity *ρ* according to the formula *ρ = RS*/*L* (where *S* is the cross-sectional area of the Cu strip and *L* is the length of the Cu strip). The tensile strength *R_m_* and elongation ε after the break of the material is calculated according to the following equations: *R_m_* = *F_m_*/*S_o_* and *ε* = (*L_u_* − *L_o_*)/*L_o_.* Where *F_m_* is the maximum force during stretching, *S_o_* is the original cross-sectional area, *L_u_* is the total length after fracture, and *L_o_* is the original length [29]. Finally, metallographic samples are made to observe the evolution of microstructure and morphology.

**Figure 3:**
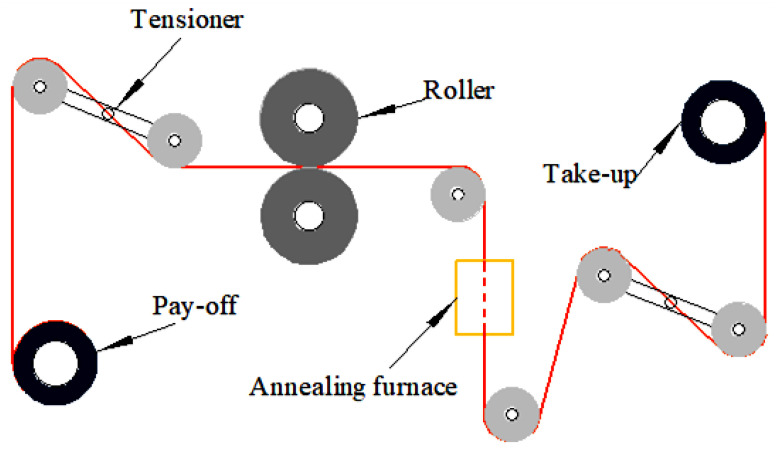
Cu strip manufacturing flow chart.

The Cu strips produced by 60% reduction rate rolling of the Cu wire of φ57 µm Specification 1 are annealed in 1000 mm long quartz tube at 200 °C, 250 °C, 300 °C, 350 °C, 400 °C, 450 °C, 500 °C and 550 °C respectively (annealing speed is 30m/min and tension is 8 g). The temperature fluctuation range is ±1 °C, and high-purity N_2_ protection is used to prevent the Cu strip from oxidizing at high temperatures during annealing. The tension of the Cu strip is controlled by a couple of swing bar-type balance devices with a tension accuracy of 0.1 g [30]; the process is shown in Figure 3.

The Cu strips produced by the above rolling are annealed at the speed of 2 m/min, 10 m/min, 20 m/min, 30 m/min, 40 m/min, 50 m/min, and 60 m/min respectively (the annealing temperature is 300 °C and the annealing tension is 8 g). The tensile strength, elongation, and resistance of the above Cu strips were measured, and metallographic samples were prepared. The variation rule of performance and micro-morphology of Cu strip at different annealing temperatures and different annealing speeds were studied. The Cu strips produced by 64% reduction rate rolling of the Cu wire of φ35 µm Specification 1 was studied about the annealing tension test (annealing temperature is 300 °C and annealing speed is 20 m/min). The pre-tension of 2 g, 4 g, 6 g, 8 g, and 10 g was used, respectively, during annealing, and then surface morphology was observed by sampling. The effect of tension on the quality of the Cu strip during annealing was studied.

The metallographic test procedure is as follows, cut a certain length of a Cu strip, put it in the bottom of the rubber mold, and fill it with the mass ratio A:B = 3:1 liquid epoxy resin, remove it from the rubber mold when it solidifies naturally after waiting for a while. Then grind these samples with 200#, 400#, 600#, 800#, and 1000# SiC sandpaper in a turned-on grinding and polishing machine and keep the water flowing during grinding. Each grinding level requires that the scratches from the last sandpaper grinding be completely eliminated. The metallographic samples have a nearly smooth surface after being ground with 1000# SiC sandpaper. These samples were then finely polished with 1.0 µm and 0.5 µm diamond polish until the surface of the sample was free of obvious scratches under metallographic microscope observation. The polished samples were coated with corrosives prepared with 5 g Cu chloride and 100 mL ammonia aqueous solution for 5–8 s, and then the surface of the samples was washed with distilled water and anhydrous ethanol successively. Finally, dry the absolute alcohol. The microstructure of the Cu strip was observed by SEM (scanning electron microscope) after gold spraying on the surface of the sample. Another way is the Cu strip was cut by FIB (focused ion beam), and then the grain size and distribution of the section were observed and counted by SEM.

## 3. Results

### 3.1. Study on the Influence of Rolling Process on Surface Quality and Properties of Bonding Cu Strips

**Figure 4:**
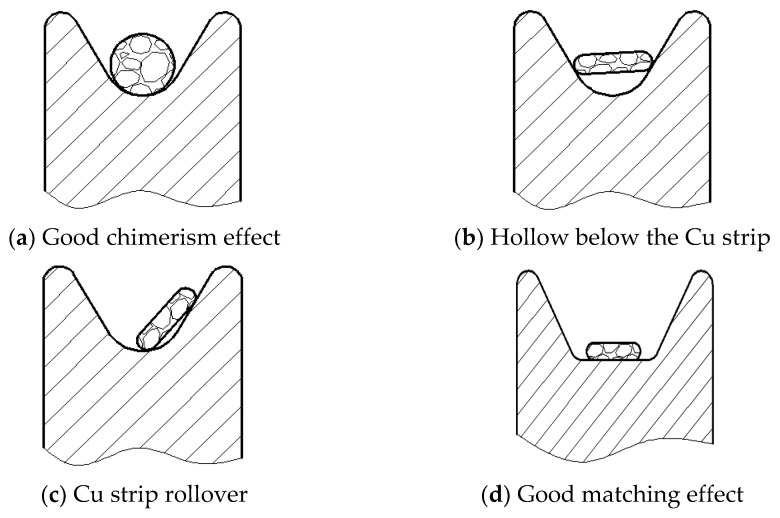
Matching condition of guide wheel with Cu wire/strip.

**Figure 5:**
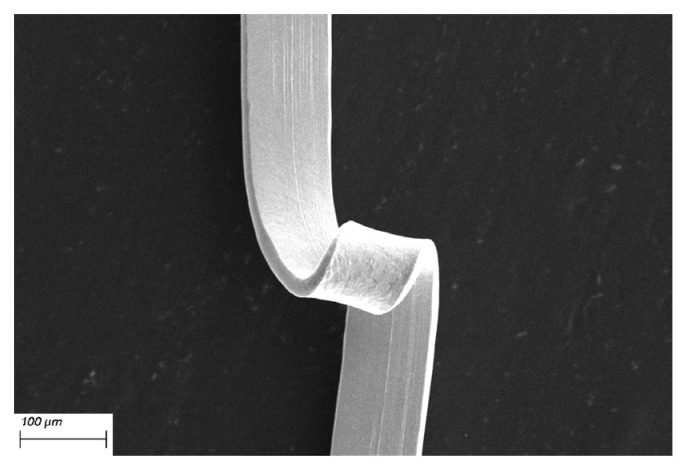
Torsion of bonding Cu strip.

The traditional bonding wire is usually produced by drawing, so the cross-section is circular, which often matches V-grooved guide pulleys with good stability for circular cross-section Cu wires, as shown in Figure 4a. However, the cross-section of the bonding Cu strip is “drum-shaped” (or approximate rectangle) and has a certain thickness and width. When cooperating with the V-guide pulley, it is easy to make the Cu strip hollow underneath or the Cu strip rubs against the side of the groove of the guide pulley, resulting in surface scratches, as shown in Figure 4b,c, which is easy to make, Cu strips tend to kink and affect the application, such as in Figure 5. Therefore, the grooved flat guide is used in the production, and the Cu strip fits well with the bottom of the guide pulley and avoids bending the strip, as shown in Figure 4d. In addition, all guide pulleys need to be installed in the same plane to prevent misalignment of adjacent guide pulleys that could cause kinking of the Cu strip during the winding process.

### 3.2. Effect of Rolling on Mechanical Properties and Microstructure of Bonding Cu Strips of Different Specifications

**Figure 6:**
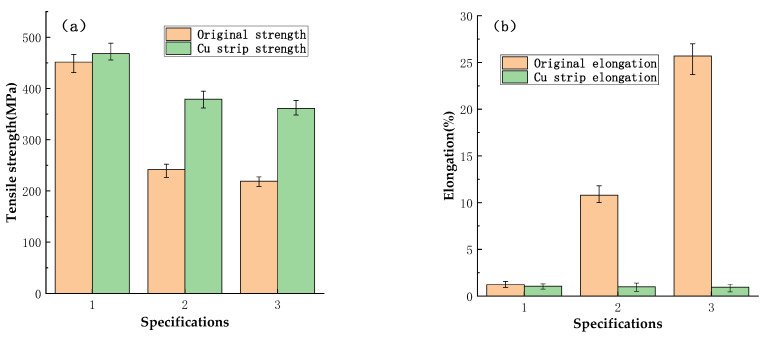
Changes in properties of bonding Cu wire before and after rolling ((**a**) tensile strength; (**b**) elongation).

The variation of properties of Cu wires of different specifications after rolling at the same reduction rate is shown in Figure 6. Because there are many dislocations accumulation and lattice defects in the Cu wire of specification 1, which is difficult to increase again after rolling, so the trend of strength increase is not obvious, and the elongation hardly changes, as shown in Figure 6b.

**Figure 7:**
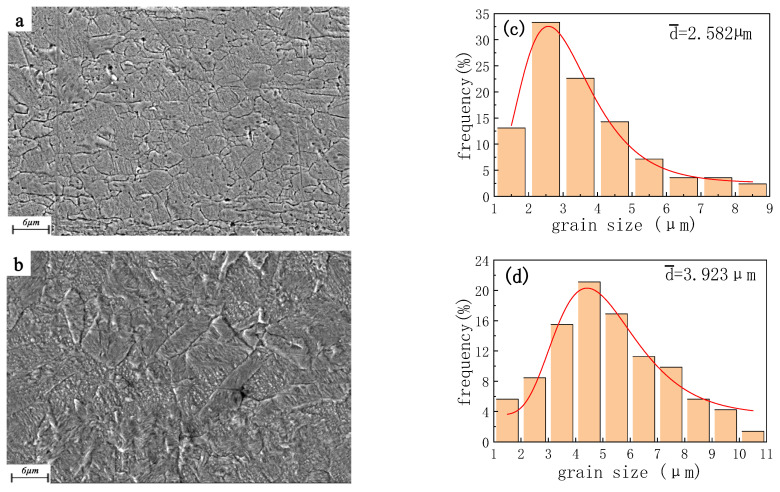
Microscopic morphology and grain size of Cu wire/Cu strip ((**a**,**b**) are Cu wire/Cu strip; (**c**,**d**) are corresponding grain size statistics).

However, the dislocation of specifications 2 and 3 Cu wires increases sharply after rolling, and the phenomenon of work hardening reappears, so the strength increases obviously, which increases from 241.8 MPa and 219.1 MPa to 379.0 MPa and 360.9 MPa, respectively. At the same time, the elongation decreases greatly with a reduction of 90% and 95.8%, respectively. Both of them showed a brittle fracture in a tensile test. Plastic deformation of metal is achieved by dislocation movement. The Cu strip is rolled to produce a large number of dislocations, whose increased dislocation density and enhanced interaction act as a barrier to lattice slip, quickly leading to an increase in metal strength and hardness. Figure 7 shows the micro-morphology observed from ND and corresponding grain size changes of φ57 µm specification 3 Cu wire before and after rolling. Because the annealed sample has a large grain size and less surface constraint, it is easier to deform under pressure. The original equiaxed grains extend along the TD and RD after rolling and show a flattened shape in ND, and the average size of the grains increases from 2.582 µm to 3.923 µm.

In addition, Figure 6 also showed an interesting phenomenon that the properties of Cu wires treated under different heat treatment conditions are almost identical after rolling under the same conditions, which indicates that rolling can strongly and effectively change the mechanical properties of Cu strips.

### 3.3. Effect of Different Reduction Rates on Microstructure of Bonding Cu Strips

**Figure 8:**
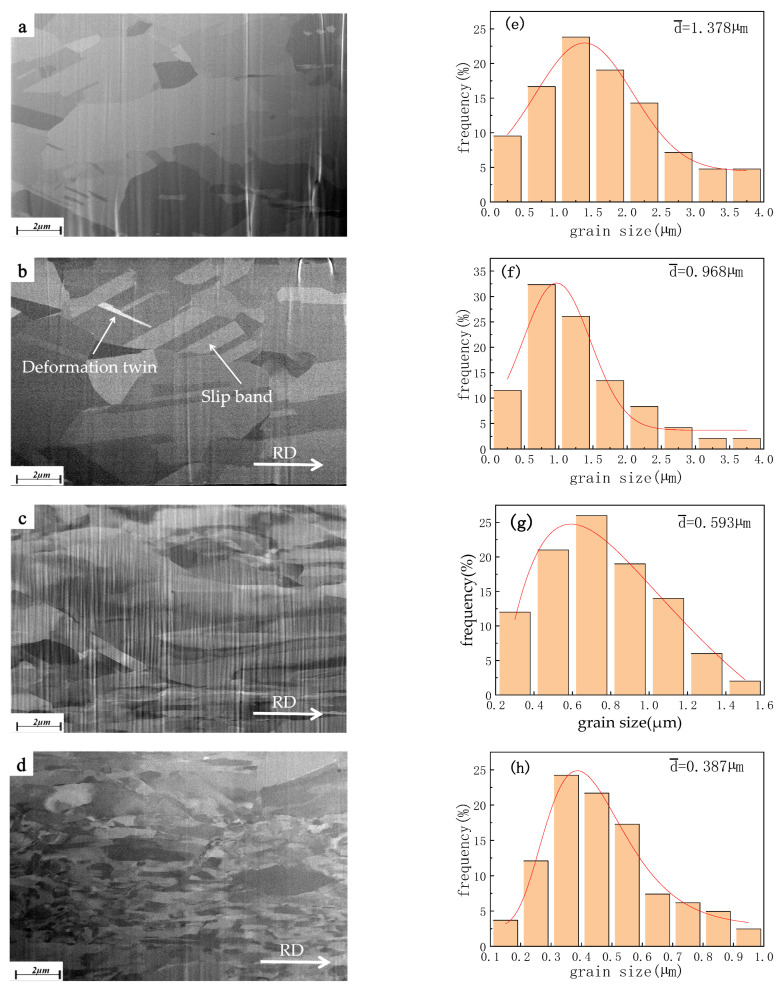
Microstructure of Cu strips at different compression rates. ((**a**) Original state, (**b**) 40%, (**c**) 60%, (**d**) 80%; (**e**–**h**) are corresponded grain size distribution).

Figure 8 shows the image obtained from the TD under SEM observation of the sample after cutting by FIB. The microstructure of the original Cu wire of φ57 µm specification 2 consists mainly of homogeneous equiaxed grains, and that average size is 1.378 µm. as shown in Figure 8a,e. When the reduction rate is 40%, the grain slip and extend in the RD, accompanied by some large-angle grain breakage. The deformation twins, slip lines and slip zones, obviously appear, but the overall deformation is small, and the average grain size is 0.968 µm, as shown in Figure 8b,f. When the reduction ratio is 60%, the original coarse grain starts to crush into finer sub-grains, the grain boundary becomes blurred, and the grains in the RD are elongated while the size in TD continues to decrease, as shown in Figure 8c. The reduction rate continues to increase to 80%, the grain is pulled longer in RD, and the grain boundary becomes more blurred, showing an approximate fibrous texture [31] in TD, and the average grain size is 0.387 µm, as shown in Figure 8d,h.

In addition, small defects (small pits, fine scratches, etc.) on the surface of the Cu wire can be effectively eliminated and become smoothed out by the press rolling. Therefore, rolling has certain tolerance to the surface quality of Cu wire, and it is an excellent strip processing method.

### 3.4. Effect of Different Reduction Rates on Mechanical and Electrical Properties of Bonding Cu Strips

**Figure 9:**
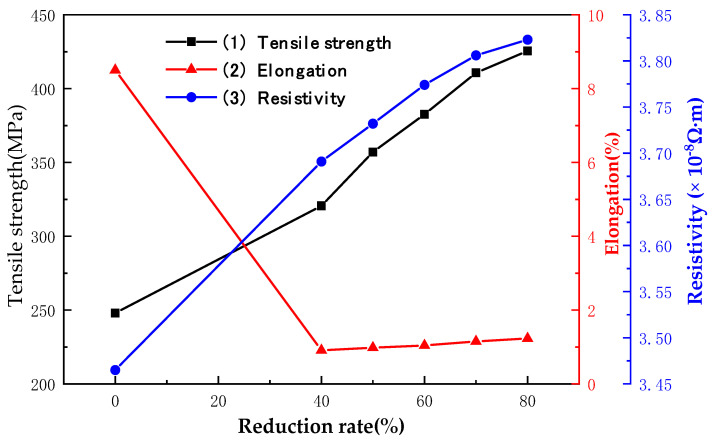
Mechanical and electrical properties of bonding Cu strips at different reduction rates.

Figure 9 shows the Mechanical and electrical properties of φ57 µm bonding Cu wire at different reduction rates. The mechanical property improvement of rolled Cu strip was due to the grain refinement and the increase in dislocation density [32]. The tensile strength and elongation of the original bonding Cu wire are 248.0 MPa and 8.50%, respectively. The lattice defects, such as the dislocation density of bonding Cu wires, increase greatly during rolling, and severe plastic deformation occurs in the grains. With the increase of the reduction rate, the degree of grain deformation increases, and the dislocation plugging becomes more serious. A large number of dislocations accumulated at the subgrain boundary will hinder the movement of dislocations and reduce the metal plastic deformation ability. When the reduction rate is 40%, the elongation of the Cu strip drops rapidly to 0.91% with a decrease of 88.7%, while the tensile strength increases to 320.6 MPa by 29.2%. A greater external force is required if the metal is to continue to deform plastically. Therefore, the bonding Cu strip has higher hardness and strength after rolling, resulting in stronger deformation resistance. The tensile strength increased to 425.5 MPa as the reduction rate was 80%.

Material strength has a certain relationship with grain size, which is a Hall–Petch relation [33]:

(1) $$\sigma_{y}=\sigma_{o}+Kd^{-\frac{1}{2}}$$

where *σ_y_* is the yield strength of the material, *σ_o_* is friction resistance, *K* is yield constant, and *d* is average grain size.

It can be seen from the above formula that the strength of the material should increase with the decrease in grain size. However, when the grain size is reduced to a certain extent, the change of material strength will deviate, the anti-Hall-Petch relationship will occur, and the properties will change greatly [29]. The elongation of the Cu strip increases slightly in the range of a 40–80% reduction rate. Because severe dislocations formed by plastic deformation tend to move to annihilate at grain boundaries as the specific surface area increases when the thickness of the Cu strip is less than a certain degree. Although new dislocations will occur after continuous rolling, the consumption rate of dislocations inside the Cu strip is greater than the growth rate at this time, resulting in certain softening of the material [34], as shown in Figure 9 Curve (2). The maximum reduction rate in this experiment is 80%, the grain size cannot reach the superfine grain level, and the softening degree of the Cu strip is not enough to offset the degree of work hardening, so the elongation rate has a small raise and the tensile strength is still increasing.

The resistivity of metal is mainly composed of phonon, dislocation, point defect (soluble atoms, impurities, vacancies, etc.), and the scattering effect of electrons at the grain interface, etc. [35] because the Cu strip of high purity, dislocation, and grain boundary are the main factors affecting its resistivity.

As shown in Figure 9, curve 3, the overall trend of resistivity of the Cu strip increases approximately linearly with the reduction rate rise. The Cu wire without rolling has fewer internal defects, a larger grain size, a small proportion of inner grain interface area per unit volume, small scattering effect of grain boundary on electrons, so it has a lower resistivity (3.46 × 10^−8^ Ω·m). Dislocation density and lattice defect increased after rolling of the Cu strip, and block grains gradually broke down, resulting in a large number of sub-grain boundaries. As the reduction rate increased and the deformation degree of the Cu strips scaled up, more lattice defects occurred, the grain boundary ratio increased, and the more obstruction the electrons were subjected to when passing through the Cu strip. The resistivity increases to 3.81 × 10^−8^ Ω·m when the reduction rate reaches 80%.

### 3.5. Effect of Heat Treatment Temperatures on Microstructure of Bonding Cu Strip

The phenomenon of work hardening and residual stress produced by rolling is not conducive to direct application, so annealing is necessary to eliminate the above defects and improve the mechanical and electrical properties and microstructure of the Cu strip.

**Figure 10:**
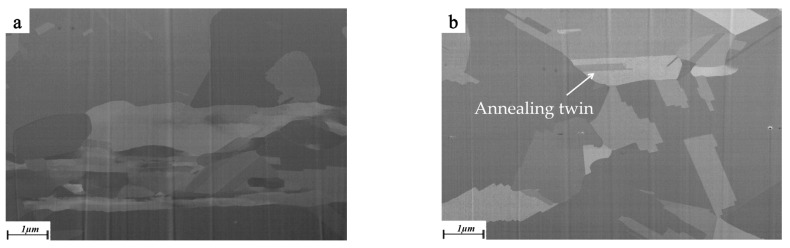

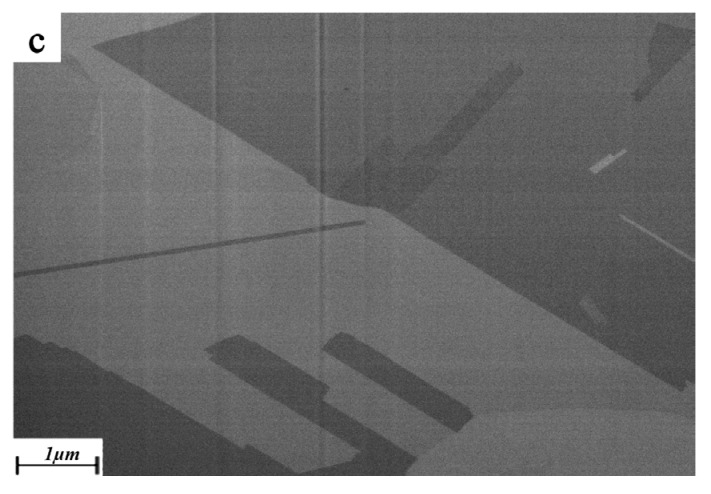
Microstructure morphology of Cu strip at different annealing temperatures ((**a**) 300 °C, (**b**) 400 °C, (**c**) 500 °C).

The microstructure of the Cu strip without annealing treatment is uniform and slender, as shown in Figure 8c. When the annealing temperature is 300 °C, the movement of point defects and line defects in the brass ribbon fiber tissue eliminates a large number of dislocations, and lattice defects are greatly absorbed. The fiber structure begins to separate and coarsen with several large angle grains appearing, which have a tendency to transform into equiaxed grains, as shown in Figure 10a. When the annealing temperature reaches 400 °C, the release tendency of deformation energy storage of the Cu strip is strengthened, lattice defects such as dislocation are further reduced, and severe deformation of Cu strip surface provides favorable conditions for recrystallization, thus accelerating recrystallization behavior [36]. The grain shape is obviously coarsened, and new grain boundaries gradually form. Annealed twins also begin to appear when recrystallized grains are produced, and the deformation structure disappears completely, as shown in Figure 10b. At an annealing temperature above 400 °C, the fiber texture of the Cu strip completely disappears and redistributes and gradually evolves into short strip or block grains. Due to the increase of recrystallized scale and grain size, the migration of grain boundaries increases, and the grains begin to grow thicker and bigger, as shown in Figure 10c.

### 3.6. Effect of Heat Treatment Temperature on Mechanical and Electrical Properties of Cu Strip

**Figure 11:**
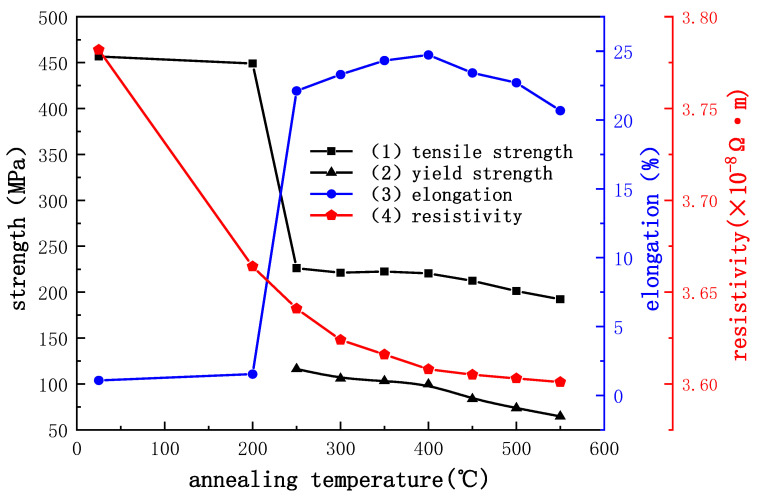
Effect of heat treatment temperature on various properties of Cu strip.

The performance curves of bonding Cu strip at different annealing temperatures are shown in Figure 11. The bonding Cu strip anneals at 200 °C with only a small amount of recovery, eliminating a small part of the dislocations and work hardening due to cold processing. The tensile strength and elongation of the Cu strip are almost unchanged from the original state. When the annealing temperature reaches 250 °C, the Cu strip begins to recover, and the work hardening is largely eliminated. The tensile strength decreases sharply to 226.1 MPa, which is 50.5% lower than the original state, while the elongation rises rapidly to 22.1%. With the annealing temperature increased to 400 °C, the bonding Cu strip completely recovers and begins to recrystallize, the elongation increases slowly to 24.7%, the strength decreases slightly, and the work hardening disappears. The grain size becomes larger when the annealing temperature is 550 °C. Coarse grains reduce the hindrance to dislocation movement, which makes the Cu strip more prone to stress concentration during tension, resulting in reduced mechanical properties, elongation, and tensile strength to 192.2 MPa and 20.7%, respectively.

Yield strength is an important index for evaluating material properties. High yield strength means good shape retention and is not easily deformed in service. However, excessive yield strength also means that the material has a higher hardness, which is relatively difficult to bond or easily destroy the substrate, while bonding with low yield strength is prone to collapse and deformation.

**Figure 12:**
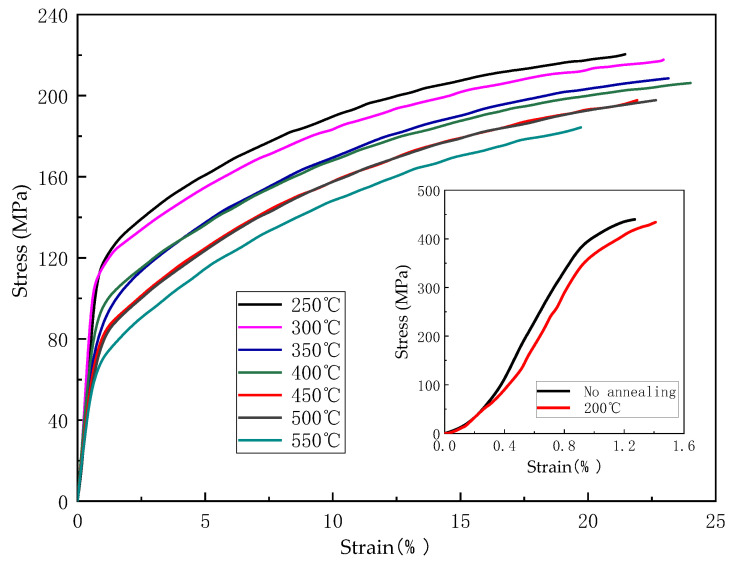
Cu strip stress-strain curves at different annealing temperatures.

From Figure 12, it can be seen that the tension curve of annealed Cu strip after 200 °C is a little different from the unannealed one; both of them were without obvious yield stage and large plastic deformation and showed a brittle fracture in a tensile test. This is because the recovery of the Cu strip at 200 °C is too low to soften the material, so it cannot bond directly. When the annealing temperature exceeds 200 °C, lattice defects such as dislocations inside the Cu strip are absorbed by adjacent grains during intense motion in a high-temperature field, and the growth of grains decreases its hardness while increasing its plastic deformation ability. The stress decreases with the increase of grain size [37], and the tensile curve in Figure 12 shows an obvious elastic and yield phase. Based on the limited hardening mechanism of dislocation sources, the decrease of lattice defects results in the decrease of Cu strip strength and the increase of elongation [38,39,40]. In addition, the decrease in dislocation density caused by annealing may lead to an increase in the amount of slip during deformation, thus accelerating the improvement of plasticity [41]. Compared with the Cu strip without annealing, its elongation can be increased by more than 20 times.

Resistivity is an important criterion for evaluating the electrical properties of bonding materials. The resistivity of metal is mainly caused by a phonon, dislocation, point defects (soluble atoms, impurities, vacancies, etc.), and the scattering effect of the interface on electrons. The resistance of high-purity Cu is mainly determined by dislocation defects and grain boundaries. Cu strips have higher resistivity because of the increased proportion of dislocations and grain boundaries produced by rolling. For bonding Cu strips, the lower the resistivity, the larger the current that can be carried. Heat treatment is an effective means to reduce the resistivity of metal materials.

The influence of annealing temperature on the electrical properties of Cu strips is shown in Figure 11, curve 4. The unannealed Cu strip has a large number of lattice defects, such as dislocations and high-density grain boundaries formed from the breakage of massive grains, which results in a high resistivity of the Cu strip (3.78 × 10^−8^ Ω∙m). After annealing, the deformed storage energy of the Cu strip is released, the atoms move vigorously in the high-temperature field and tend to arrange orderly and periodically accompanied by the lattice defects such as dislocation are greatly reduced. Therefore, the resistivity descends gradually as the resistance of the electron motion in the Cu strip decreases. The resistivity of the Cu strip decreases to 3.62 × 10^−8^ Ω∙m at an annealing temperature of 300 °C. When the annealing temperature is higher than 400 °C, dislocation and other lattice defects are completely eliminated, and the grains grow gradually. Free electrons are less affected by the scattering of the grain boundaries when they move, but their contribution to the reduction of resistivity is limited, so the trend of resistivity decline is more gentle. Cu strip resistivity is 3.60 × 10^−8^ Ω∙m when the annealing temperature is 550 °C.

### 3.7. Effect of Annealing Speed on Mechanical Properties of Cu Strip

**Figure 13:**
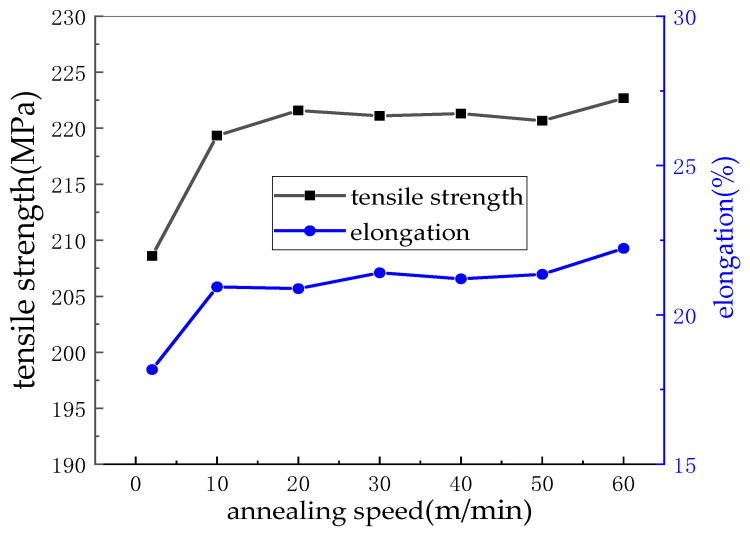
Effect of annealing speed on mechanical properties of Cu strip.

Heat treatment is the last process in the production of bonding Cu strips. The slower rate makes the Cu strip softer due to the longer annealing time, but it also loses some strength. Faster speed means higher productivity, but the annealing time reduced maybe result in the hardening work not being completely eliminated. The effect of annealing temperature on the mechanical properties of bonding the Cu strip is shown in Figure 13.

At a speed of 2 m/min, the Cu strip recrystallized for a long time in the annealed tube, and the grain grew, which reduced the mechanical properties; both the tensile strength and elongation were low, 208.6 MPa and 18.7%, respectively. However, there is no significant trend in the tensile strength and elongation of the Cu strip during the annealing speed of 10–50 m/min. Because at this speed range, the Cu strip is able to recover and complete the elimination of work hardening, and the recrystallization phenomenon is slight, so the performance is stable. When the speed is increased to 60 m/min, the Cu strip has a slightly lower recovery and remains partially hardened due to its shorter residence time in the annealed tube, resulting in a relatively high tensile strength and elongation, which is 222.67 MPa and 22.23%, respectively.

### 3.8. Effect of Annealing Tension on Surface Quality of Cu Strip

**Figure 14:**
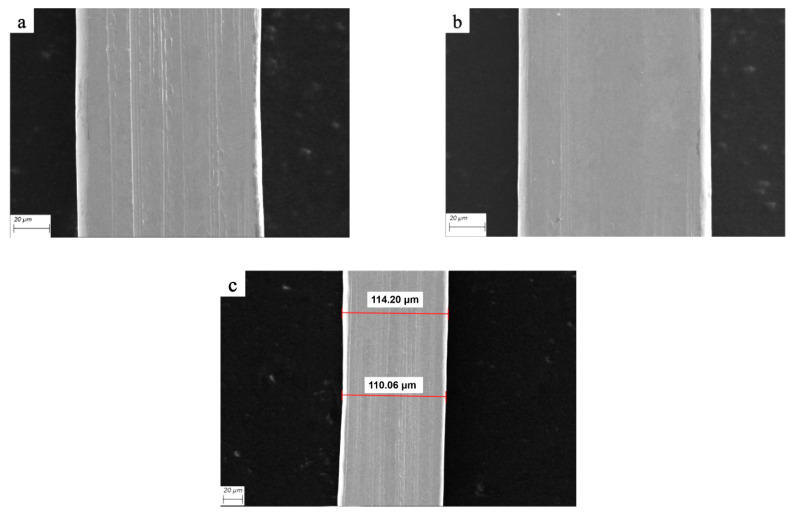
Surface quality of Cu strips under different annealing tensions ((**a**) surface mechanical damage, (**b**) surface smoothness, (**c**) non-uniform dimensions).

When pre-tension is 2–4 g during annealing, bonding Cu strip in the annealing tube is susceptible to strong shaking due to interference from blowing of protective gas, and easy to scratch the surface of the Cu strip by reason of in contact with the annealing tube nozzle, as shown in Figure 14a. When 6–8 g tension is applied, there is no obvious shaking during the annealing of the bonding Cu strip, and the surface is smooth and without scratches, as shown in Figure 14b; the annealing tension is 10 g due to the sliding motion of grains caused by the higher tension. The plastic deformation of the Cu strip in the thermo-force coupling field results in dimensional inconsistency, which affects the application, as shown in Figure 14c. For Cu strips rolled with φ35 µm Cu wire, the annealing tension range is optimal at 6–8 g.

## 4. Conclusions

(1) With the increase of the reduction rate to 80%, the coarse grains are gradually broken and refined, and the Cu strip structure is flat in the ND direction. The increase in dislocation density hinders the movement and slip of dislocations, resulting in an increase in the tensile strength of the bonding Cu strips from 248.0 MPa to 425.5 MPa, and a sharp decrease in elongation from 8.50% to 0.91%. The Cu strip microsoftens, and its elongation increases slightly to 1.23% due to the high reduction rate. The increase of lattice defects and grain boundary density results in an approximately linear increase of resistivity with the increase of the reduction rate (from 3.46 × 10^−8^ Ω∙m to 3.81 × 10^−8^ Ω∙m).
(2) As the annealing temperature rises to 400 °C, the Cu strip recovers, and dislocation is eliminated at high temperatures. The softening of the Cu strip results in the decrease of tensile strength from 456.66 MPa to 220.36 MPa and the increase of elongation from 1.09% to 24.73%. The growth of grain reduced the tensile strength and elongation to 192.2 MPa and 20.68%, respectively, when the annealing temperature rises to 550 °C. The changing trend of yield strength is the same as the tensile strength of the bonding Cu strip. In the annealing speed range of 10–50 m/min, the strength and elongation of the Cu strip are nearly unchanged, and its performance is stable.
(3) The resistivity of the Cu strip decreases considerably during 200–300 °C annealing temperature because the lattice defects are largely eliminated (from 3.78 × 10^−8^ Ω∙m reduced to 3.62 × 10^−8^ Ω·m). Recrystallization occurs slightly at annealing temperature during 300–400 °C, and the decreasing trend of resistivity is slowed down. When the annealing temperature is higher than 400 °C, the grains begin to grow, and the resistivity decreases more smoothly. The optimum annealing tension range is 6–8 g. Low tension will loosen the Cu strip and cause burns, while high tension will narrow the Cu strip width.

## Figures and Tables

**Table 1:** Thick Cu wire.

φ57 µm Cu Wire	Elongation (%)	Tensile Strength (MPa)
Specification 1	0.92	448.57
Specification 2	10.8	236.96
Specification 3	25.7	214.68

**Table 2:** Thin Cu wire.

φ35 µm Cu Wire	Elongation (%)	Tensile Strength (MPa)
Specification 1	0.84	419.66
Specification 2	9.42	230.20
Specification 3	21.95	215.94

**Table 3:** Test Equipment and Model.

Equipment	Model
High Precision Rolling Mill	M13 + E100 + AF0 + R5
On-line Heat Treatment Equipment	YK-450
Scanning Electron Microscope	JEOL JSM-6700F
Strength Tester	E42.503
Resistivity Tester	HIOKI3226

## References

[1] Jie W., Rockey T., Yauw O., Shen L., Chylak B. Bonding of Ag-alloy wire in LED packages. Proceedings of the Electronic Manufacturing Technology Symposium IEEE.

[2] Liqun G., Qiang C., Juanjuan L., Zhengrong C., Jianwei Z., Maohua D., Chung M. (2013). Comparison of Ag wire and Cu wire in memory package. Funct. Mater. Devices.

[3] Tian F. (2016). Design and Research of Visual Positioning Technology Based on Chip Lead Bonding.

[4] Xu Q., Zhu Z., Huang Y. (2021). Analysis of Impact of Contamination on Reliability of Raw Material Enclosures for Integrated Circuits. Environ. Technol..

[5] Schneider-Ramelow M., Ehrhardt C. (2016). The reliability of wire bonding using Ag and Al. Microelectron. Reliab..

[6] Chuang T.-H., Lin H.-J., Chuang C.-H., Shiue Y.-Y., Shieu F.-S., Huang Y.-L., Hsu P.-C., Lee J.-D., Tsai H.-H. (2014). Thermal stability of grain structure and material properties in an annealing twinned Ag–4Pd alloy wire. J. Alloys Compd..

[7] Lin L., Zang X.D. (2014). Introduction of a Corrosion-resistant High-reliability Silver Alloy Bonding Wire in Package. Electron. Packag..

[8] Tang R., Yang X.L., Wu B.A., Tang H.Y., Zhang C. (2019). Analysis and prospect on alloy system and compositions of gold-silver alloy bonding wire from the perspective of patent citations. Electron. Compon. Mater..

[9] Li X.W., Cai J.L., Chen L., Lin J. (2019). Analysis and comparison of performance of bonding wire in LED packaging. China Light Light..

[10] Hsueh H.-W., Hung F.-Y., Lui T.-S., Chen L.-H. (2011). Microstructure, electric flame-off characteristics and tensile properties of silver bonding wires. Microelectron. Reliab..

[11] Zhong M.J., Huang F.X., Yuan H.G. (2017). Research progress on the copper and silver bonding wire materials. Mater. Rep..

[12] Gu B., Shen S., Li H. (2022). Mechanism of microweld formation and breakage during Cu–Cu wire bonding investigated by mo-lecular dynamics simulation. Chin. Phys. B.

[13] Zhang H., Guo Z.N., Li Y.C., Chen Y.J. (2011). Research progress of fine copper wire bonding technology. Proceedings of the 14th National Special Processing Academic Conference.

[14] He X., Guo L., Gaosen G., Fengling S., Zhu D. (2022). Effects of different inhibitor on antioxidation of copper bonding wire at room temperature. J. Mater. Sci. Mater. Electron..

[15] Liu P., Tong L., Wang J., Shi L., Tang H. (2011). Challenges and developments of copper wire bonding technology. Microelectron. Reliab..

[16] Chauhan P.S., Choubey A., Zhong Z., Pecht M.G. (2014). Copper Wire Bonding.

[17] Zhong Z.W. (2011). Overview of wire bonding using copper wire or insulated wire. Microelectron. Reliab..

[18] Hamid K.A., Badarisman A.H., Jalar A., Abu Bakar M. (2022). Effects of electrolyte towards copper wire metallurgical interconnection in semiconductor. J. Phys. Conf. Ser..

[19] Lu K., Ren C.L., Gao N.Y., Ding R.Z. (2010). The Process and Reliability Researches of Copper Wire Bonding. Electron. Packag..

[20] Gan C.L., Ng E.K., Chan B.L., Classe F.C., Kwuanjai T., Hashim U. (2013). Wearout Reliability and Intermetallic Compound Diffusion Kinetics of Au and PdCu Wires Used in Nanoscale Device Packaging. J. Nanomater..

[21] Fischer A.C., Korvink J., Roxhed N., Stemme G., Wallrabe U., Niklaus F. (2013). Unconventional applications of wire bonding create opportunities for microsystem integration. J. Micromech. Microeng..

[22] Ani F.C., Aziz M., Jalar A., Abdullah M.Z., Rethinasamy P. Effect of gold concentration through a single dynamic wave soldering process. Proceedings of the IEEE International Conference on Electronic Materials & Packaging.

[23] Breach C.D., Lee T.K. (2012). Shear Strength and Failure Modes of As-Bonded Gold and Copper Ball Bonds on Aluminum Metalli-zation. J. Electron. Mater..

[24] Appelt B.K., Tseng A., Huang L., Chen S. Is copper wire bonding ready for automotive applications?. Proceedings of the 2011 IEEE 13th Electronics Packaging Technology Conference.

[25] Alim A., Abdullah M., Aziz M.A., Kamarudin R. (2021). Die attachment, wire bonding, and encapsulation process in LED packaging: A review. Sens. Actuators A Phys..

[26] Murali S., Srikanth N., Vath C.J. (2003). An analysis of intermetallics formation of gold and copper ball bonding on thermal aging. Mater. Res. Bull..

[27] Leong H.Y., Yap B.K., Khan N., Ibrahim M.R., Tan L.C., Faiz M. (2014). Characterisation of insulated Cu wire ball bonding. Mater. Res. Innov..

[28] (2022). Gold-Based Bonding Wire and Bandlet for Semiconductor Package.

[29] Kuang Y., Ma J. (2000). Microelectronic packaging technology towards the new century. Electron. Technol..

[30] Zhao H., Wang J. (2010). Couple Balance Swing Rod Tension Balancing Device.

[31] Goto M., Han S., Yakushiji T., Lim C., Kim S. (2006). Formation process of shear bands and protrusions in ultrafine grained copper under cyclic stresses. Scr. Mater..

[32] Dong Z., Fei X., Gong B., Zhao X., Nie J. (2021). Effects of Deep Cryogenic Treatment on the Microstructure and Properties of Rolled Cu Foil. Materials.

[33] Delin S. (2003). Mechanical Properties of Engineering Materials.

[34] Wan Y., Chang S., Song M., Duo Y., Li J., Liu X. (2018). Microscale effect of industrial pure copper ultra-thin strip rolling. Met. Heat Treat..

[35] Hong S.I., Hill M.A. (1999). Mechanical stability and electrical conductivity of Cu–Ag filamentary microcomposites. Mater. Sci. Eng. A.

[36] Li X., Jiang G., Di J., Yang Y., Wang C. (2019). Effect of cryogenic rolling on the microstructural evolution and mechanical properties of pure copper sheet. Mater. Sci. Eng. A.

[37] Zhao J., Huo M., Ma X., Jia F., Jiang Z. (2019). Study on edge cracking of copper foils in micro rolling. Mater. Sci. Eng. A.

[38] Huang X., Hansen N., Tsuji N. (2006). Hardening by Annealing and Softening by Deformation in Nanostructured Metals. Science.

[39] Wei Z., Yao S., Ning Z., Huang X., Wang J., Tang G., Shan A. (2012). Rapid hardening induced by electric pulse annealing in nanostructured pure aluminum. Scr. Mater..

[40] Kamikawa N., Huang X., Tsuji N., Hansen N. (2009). Strengthening mechanisms in nanostructured high-purity aluminium deformed to high strain and annealed. Acta Mater..

[41] Li Z., Fu L., Fu B., Shan A. (2012). Effects of annealing on microstructure and mechanical properties of nano-grained titanium produced by combination of asymmetric and symmetric rolling. Mater. Sci. Eng. A.

